# Common Carotid Artery Diameter and Cardiovascular Risk Factors in Overweight or Obese Postmenopausal Women

**DOI:** 10.1155/2012/169323

**Published:** 2012-08-21

**Authors:** Kelly D. Lloyd, Emma Barinas-Mitchell, Lewis H. Kuller, Rachel H. Mackey, Eric A. Wong, Kim Sutton-Tyrrell

**Affiliations:** ^1^Department of Epidemiology and Public Health, School of Medicine, University of Maryland Baltimore, 660 W. Redwood Street, Baltimore, MD 21201, USA; ^2^Department of Epidemiology, Graduate School of Public Health, University of Pittsburgh, 130 DeSoto Street, Pittsburgh, PA 15261, USA; ^3^Department of Emergency Medicine, Kaiser Permanente Fremont Medical Center, 39400 Paseo Padre Parkway, Fremont, CA 94538, USA

## Abstract

Arterial diameter is an underutilized indicator of vascular health. We hypothesized that interadventitial and lumen diameter of the common carotid artery would be better indicators of vascular health than carotid plaque or intima media thickness (IMT). Participants were 491 overweight or obese, postmenopausal women who were former or current hormone therapy (HT) users, 52–62 years, with waist circumference >80 cm. We evaluated cross-sectional associations of cardiovascular risk factors with carotid measures, by HT status. Former HT users had a worse cardiovascular profile than current HT users: larger adventitial (6.94 mm versus 6.79 mm) and lumen diameter (5.44 mm versus 5.31 mm, both *P* < 0.01) independent of cardiovascular risk factors; IMT and plaque were similar. Larger diameters were best explained by former HT use, higher pulse pressure, and greater weight. Independent of potential confounders, overweight and obese postmenopausal former HT users had larger carotid diameters than current HT users. Carotid diameter should be considered in studies of HT.

## 1. Introduction

Until recently, the adventitia has been largely neglected [1, 2]. The adventitia is more than just the outermost layer of the artery; it is now known to play a critical role in vascular remodeling and other important processes of the artery [1–3]. Recent attention to the role of the adventitia in vascular remodeling has increased reporting of common carotid artery interadventitial diameter (IAD), a noninvasive measure of vascular geometry and health.

The results from one of these studies, an ancillary of the Study of Women's Health Across the Nation (SWAN) called SWAN Heart, suggest that declining endogenous estrogen that accompanies the menopausal transition has a direct effect on the peripheral vasculature [4]. The study found lower levels of estradiol were significantly associated with larger common carotid artery IAD even after adjustment for cardiovascular risk factors [4]. Other studies have shown that larger IAD is associated with increasing age [5–7], cardiovascular risk factors [4–6, 8–13], prevalent cardiovascular disease [12, 14, 15], and incident cardiovascular events [15]. Thus, the increase in IAD observed with declining endogenous estrogen [4] suggests that lower levels of endogenous estrogens are associated with a less healthy vasculature. 

The strong association between IAD and endogenous estrogen suggests that a similar association may exist with the use of exogenous estrogen. The purpose of this study was to determine whether postmenopausal current HT users had significantly different IAD than those who were former users of HT in the Women on the Move through Activity and Nutrition (WOMAN) randomized trial. We also wanted to determine if there were differences between other measures of vascular health.

## 2. Methods

### 2.1. Study Population and Design

This study evaluates cross-sectional associations using measurements from the baseline visit of the clinical trial (clinical trials registry number: NCT 00023543). The WOMAN trial tested the ability of nonpharmacological lifestyle intervention to modify cardiovascular risk factors in postmenopausal women. The study recruited 508 eligible African American and Caucasian women from Allegheny County, PA, between April 2002 and October 2003 through direct mailings. Eligible women were postmenopausal, between 52 and 62 years of age, able to walk, currently using HT, and willing to participate in either intervention group regardless of assignment and had a waist circumference ≥80 cm, a body mass index (BMI) between 25.0 and 39.9 kg/m^2^, blood pressure <160/95 mmHg, and low density lipoprotein (LDL) cholesterol between 100 and 160 mg/dL. Women were ineligible if they were taking medication for cholesterol, diagnosed with or on medication for diabetes, diagnosed with a psychotic disorder, or suffering from depression [16]. The results of the Women's Health Initiative estrogen/progestin arm were published in the middle of recruitment [17]; as a result the eligibility criterion of current use of HT was modified to current or recent history of hormone use [16]. Recent history of hormone use was defined as prior use of at least 2 years within 6 months of randomization. The decision to remain on HT was determined by the participant and her physician. At baseline, 40% of the women had discontinued use of HT (these women will be referred to as former HT users) and 60% remained on HT (these women will be referred to as current HT users). For those who discontinued use of HT the median time off therapy was 7 months prior to study randomization [16]. Written informed consent was obtained from all the participants. The study was approved by the University of Pittsburgh's Institutional Review Board.

### 2.2. Carotid Ultrasound Measures

**Figure 1:**
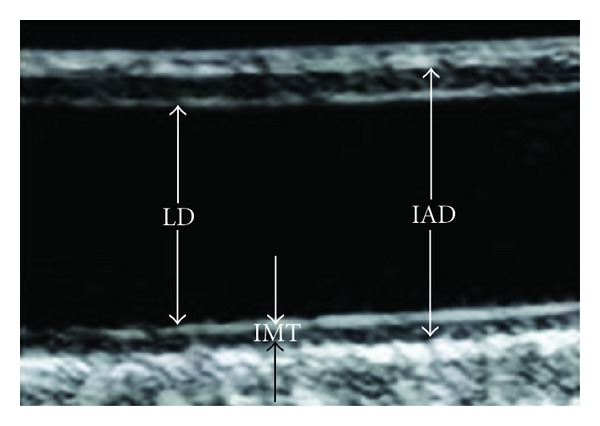
Measurement of the common carotid artery lumen diameter (LD), intima media thickness (IMT), and interadventitial diameter (IAD) also known as adventitial diameter.

Common carotid artery intima media thickness (IMT), IAD, lumen diameter (LD), and plaque were assessed by B-mode ultrasound using a Toshiba SSA-270A duplex scanner (Toshiba American Medical Systems, Tustin, CA, USA) with a 5 MHz-linear array transducer. Right and left carotid images were taken of the near and far walls of the distal common carotid artery 1 cm proximal to the carotid bulb [16]. IMT was defined as the distance from the lumen-intimal interface to the medial-adventitial interface (Figure 1). IAD was defined as the distance from the adventitial-medial interface on the near wall to the medial-adventitial interface on the far wall (Figure 1). LD was defined as the distance from the intima-lumen interface of the near wall to the lumen-intima interface of the far wall (Figure 1). Using a semiautomated edge detection software, the interfaces were traced electronically over the distal CCA and a computer generated measurement was obtained for each pixel in the area of interest; these measurements were averaged to determine IMT, IAD, and LD used for this analysis. A reproducibility study, conducted in 20 women who were similar to the women in the current study, provided an intraclass correlation of 0.98 for IMT and 0.99 for IAD [4]. The reproducibility study took place at the same lab and used the same equipment and readers as the current study.

The presence of plaque was determined for each of the 5 segments of the left and right carotid artery (distal and proximal CCA, carotid bulb, and proximal internal and external carotid artery). Plaque was defined as a distinct area protruding into the vessel lumen at least 50% thicker than the adjacent IMT. This analysis categorized plaque as either absent or present. 

### 2.3. Visits

The first of two prerandomization screening visits included a 12-hour fasting blood draw, physical measures of height, weight, waist circumference, blood pressure, the long distance corridor walk, medical, physical activity, and weight history. Conventional enzymatic methods were used to obtain total cholesterol, high density lipoprotein (HDL) cholesterol, and triglyceride concentrations from the blood samples [16]. Low density lipoprotein (LDL) cholesterol was estimated using the Friedewald equation [16]. Medical history included history of drug, vitamin/mineral supplement, and alcohol use [16]. Common carotid artery IAD, LD, IMT and plaque were measured at the second screening visit [16].

### 2.4. Analytical Methods

 Five hundred eight women were randomized into the WOMAN study. Seventeen women had incomplete data for the calculation of IAD or IMT and were excluded leaving 491 women for analysis. All analyses were completed using SAS v9.1 or v9.2 (SAS Institute Inc., Cary, NC, USA). A *P* value <0.05 was considered statistically significant.

 Descriptive statistics and normality of continuous measures were assessed for the cohort. Means and standard deviations are presented for normally distributed variables and medians and 25th and 75th percentiles are provided for nonparametric variables; dichotomous variables are presented as percents. Differences between the current HT users and the former HT users were determined using chi-square analyses for categorical variables and *t*-tests and Wilcoxon-rank sum tests for continuous variables.

 Simple linear regression was used to assess univariate associations between IAD and LD with HT and the following cardiovascular risk factors: age, race, systolic blood pressure, diastolic blood pressure, pulse pressure, BMI, weight, height, waist circumference, total cholesterol, LDL and HDL cholesterol, triglycerides, glucose, insulin, smoking status, and antihypertensive medication use. When collinearity between covariates was suspected (*r* > 0.4), the variable most strongly correlated to IAD or LD was selected for the analysis. The following variables were collinear: glucose and insulin; systolic blood pressure and pulse pressure; BMI, weight, and waist circumference; weight and height; total cholesterol and LDL. Based on Spearman correlation results glucose, pulse pressure, weight, and LDL were chosen for the multivariable models. Multivariable linear regression was used to test for the following predetermined covariates: age, race, pulse pressure, and smoking status. In addition, any statistically significant variable in the univariate analysis and any variable that differed by HT use status were also tested. Total cholesterol, HDL, LDL, glucose, and insulin differed by HT use status; addition of these variables did not alter the regression model so they are not presented in the results. The most parsimonious multivariable model was chosen.

## 3. Results

The median (25th, 75th percentiles) age of the women was 57 (55, 60) years, median BMI was 30 (28, 34) kg/m^2^; 11% were African American and 6% were current smokers. There were 197 former HT users and 294 current HT users at the time of the baseline carotid ultrasound scan. Former HT users were older and had a higher percent of African Americans (Table 1). Overall former HT users had a significantly worse cardiovascular disease risk profile than current HT users: higher total cholesterol, higher LDL cholesterol, higher glucose and insulin, and lower HDL cholesterol (Table 1). There were, however, no differences by HT status in blood pressure, measures of general or central obesity, and smoking status (Table 1).

The mean IAD was 6.94 mm for former HT users and 6.79 mm for current HT users (*P* = 0.001, Table 2). LD was also significantly larger in the former HT users than in the current HT users (5.44 mm versus 5.31 mm, *P* = 0.002, Table 2). However, IMT and presence of plaque were not different between the two groups (Table 2). 

### 3.1. Regression Results for Interadventitial Diameter

Simple linear regression showed that in addition to former HT use, larger IAD was significantly associated with greater systolic blood pressure, pulse pressure, BMI, weight, height, waist circumference (all *P* < 0.0001), glucose, insulin (both *P* = 0.001), age, Caucasian race, current nonsmoking status and use of antihypertensive medications (*P* < 0.05) (Table 3). The most parsimonious model in the multivariate analysis revealed that higher pulse pressure, higher weight and former HT use were the key factors independently associated with larger IAD. The model was also run forcing age, race, and smoking status (Table 4). In this model, hormone therapy, pulse pressure, and weight remained significantly associated with IAD (all *P* < 0.01, Table 4). The model was also run controlling for antihypertensive medication use, but this variable fell out of the multivariable model when pulse pressure was added.

Current HT use was associated with a 0.14 mm smaller IAD (Table 4). African American women had a 0.05 mm smaller IAD than the Caucasian women, although this was not significant (Table 4). Current cigarette smokers had 0.18 mm smaller IAD than current nonsmokers, with borderline significance (Table 4). 

### 3.2. Regression Results for Lumen Diameter

 Former HT use, greater BMI, weight, height, waist circumference, insulin (all *P* < 0.01), glucose, and pulse pressure (both *P* < 0.05) were associated with larger LD in univariate linear regression (Table 3). The variables that yielded the best multivariate model to explain larger LD were former HT use, higher pulse pressure, and higher weight. When age, race and smoking were forced in the model only HT use and weight remained significantly associated with LD (Table 4); the same results were seen when antihypertensive medication was added to the model (data not shown).

 Current HT use was associated with a 0.13 mm smaller LD (Table 4). Each kg of weight was associated with 0.009 mm larger LD (Table 4).

## 4. Discussion

Postmenopausal current HT users had statistically significant smaller IAD than the former HT users; this relationship remained significant after adjustment for known cardiovascular risk factors. The current HT users also had statistically significant smaller LD than the former HT users. In contrast, IMT and plaque were not statistically different between current HT users and former HT users. This suggests that HT may be associated with preserved vascular geometry in postmenopausal women. It also demonstrates the value of measuring IAD and LD in this type of study.

The adventitia, the most outer layer of the artery, is composed of supportive connective tissue, fibroblasts, collagen, and elastin fibers [1]. Estrogen is known to preserve arterial structure by slowing elastin and connective tissue degradation, and by slowing age- and estrogen-related increases in collagen which lead to increased vascular stiffening [18]. A small diameter reflects a healthy vasculature that is able to maintain an optimal balance of shear and tensile stress [19–21]. An enlarged diameter is less able to effectively control levels of shear stress. This can make the artery vulnerable to injury and atherosclerotic development [1, 11]. 

The results of the current study, specifically the association of current exogenous estrogen use with smaller IAD, is in line with the SWAN Heart study [4] that showed an association between higher levels of endogenous estrogen and smaller IAD. The current study observed a 0.15 mm difference in IAD between the current and former HT users. A longitudinal study observed 0.03 mm increase in IAD each year for women (with similar mean age and mean height as the women in the current study) [6]. So the difference in IAD observed in the current study translates to the change in IAD expected over 5 years (0.03 mm/year ×5 years = 0.15 mm). 

Current HT use was associated with smaller LD. These findings agree with the results of a cross-sectional study that found smaller LD among non-oral (percutaneous gel or transdermal patch) HT users compared to HT non-users [22]. Together, the findings from IAD and LD may suggest the positive effect of estrogen on the vasculature through maintenance of vascular structure and function. 

Both the current study and the SWAN Heart study found that larger diameter was associated with older age, higher systolic blood pressure, higher glucose, and higher insulin: all risk factors for CVD. Additional supporting evidence that enlarged diameter is an indicator of poor vascular health come from several studies showing enlarged IAD is associated with cardiovascular disease risk factors [4–6, 8–13], increased IMT [5, 12], plaque [5, 12, 23], and prevalent [12, 14, 15] and incident CVD [15]. Polak et al. recently published an article that identified a positive relationship between IAD and left ventricular mass, an indicator of left ventricular hypertrophy [13]. Each 1 gram difference in left ventricular mass was associated with 0.006 mm larger IAD in a multiethnic population of women after adjustment for height, weight, and IMT [13]. 

Arterial diameter differences in current HT users and former HT users were observed in this study but differences in IMT were not. Consistent with our findings, a cross-sectional study of an American cohort from the Atherosclerosis Risk in Communities (ARIC) study [24], found no significant difference in IMT by HT use. The women in the ARIC study were of similar age to the women in this study and also had an undefined HT regimen that was predetermined by the woman and her physician prior to the study. Selection bias may be present in both studies since women who chose to go on HT or women who chose to continue HT may have been different from the women who did not chose HT. A longitudinal study of oral therapy with one year of follow-up did not find a difference in IMT progression in HT users and non-users [25]. 

Three studies evaluated differences in IMT by HT and age [22, 26, 27]. The women were dichotomized into younger versus older (using 55 or 60 as the age cut-point). Significant differences in IMT were observed only in the older women who had longer use of HT than the younger women [22, 26, 27]. This may suggest that the effects of estrogen on IMT are evident after long-term use. Other explanations are that the differences observed are attributed to differing vascular effects of oral HT compared to transdermal HT, and fewer years on HT in the negative studies than the positive studies. One study that compared oral and non-oral therapy found transdermal HT had greater statistically significant effects on IMT than oral HT [28]. The ARIC study and this study participants used oral HT, were relatively younger and had fewer years on HT compared to the positive studies.

A limitation of this study is that the HT regimen was varied since the dose, hormone composition (estrogen only or estrogen plus progestin), and form were chosen prior to the study by the participant and her health care provider. The main type of HT was oral. One study observed that transdermal HT had greater effects on IMT than oral HT [28]. A standard dose and regimen of HT would be easier to evaluate and compare this study to previous studies. This would likely be especially true for the IMT results that were not significant in this study. Another limitation is that the adherence to HT and the level of estrogen or estradiol in the current users and former users was not assessed in this study. Although the women reported HT use we do not know their adherence rates or the level of estrogen metabolites present during the ultrasound measurements. In the future, assessment of estradiol levels should be included to improve our understanding of carotid diameter associations and dose-related effects.

Strengths of this study are that it fills a gap in the literature, the methods used are valid and reliable, the lab that performed the ultrasound measures has high quality control, and it is one of the first to evaluate IAD and HT. The contribution of the adventitia to vascular function has been largely ignored [1]. This is evident in the scarcity of literature that evaluates the measure. This study demonstrates the importance of IAD and LD as more sensitive indicators of vascular health than IMT. High resolution B-mode ultrasound is a valid and reliable detector of structural atherosclerotic changes of the arterial walls [29]. The ultrasound measures in this study were performed with excellent reproducibility (class intra correlations of 0.98 for IMT and 0.99 for IAD) and continuous quality control to ensure reliable and valid data. 

## 5. Conclusions

In conclusion, these data suggest that current HT use is associated with vascular geometry in the postmenopausal women independent of cardiovascular risk factors. It also demonstrates the importance of measuring IAD and LD in postmenopausal women with differing HT use. These measures should be included in addition to IMT to provide a more complete story of vascular response and health.

## Figures and Tables

**Table 1:** Demographic, anthropometric and health characteristics by hormone therapy use.

	Former users (*n* = 197)	Current users (*n* = 294)	*P* value^a^
Age, years	57.2 (55.2, 60.0)	56.5 (54.1, 59.2)	0.006
African American Race, %	16.9	7.2	0.008
Systolic blood pressure, mmHg	123.6 ± 13.0	123.7 ± 13.4	0.899
Diastolic blood pressure, mmHg	76.7 ± 7.6	76.1 ± 8.0	0.349
Pulse pressure, mmHg	46.8 ± 10.5	47.7 ± 10.8	0.400
Body mass index, kg/m^2^	29.9 (27.6, 33.5)	30.0 (27.5, 33.4)	0.793
Weight, kg	79.4 (72.8, 9.0)	80.7 (72.6, 89.8)	0.849
Height, cm	163.3 ± 5.6	162.9 ± 5.9	0.493
Waist circumference, cm	105.5 ± 11.8	105.9 ± 10.7	0.520
Cholesterol, mg/dL	223.1 ± 29.0	212.5 ± 26.7	<0.0001
High density lipoprotein, mg/dL	56.8 (49.7, 67.0)	59.8 (51.8, 69.0)	0.052
Low density lipoprotein, mg/dL	137.1 ± 26.0	122.1 ± 22.8	<0.0001
Triglycerides, mg/dL	119.0 (94.5, 159.0)	130.5 (96.0, 173.5)	0.222
Glucose, mg/dL	97.3 ± 9.0	93.8 ± 9.0	<0.0001
Insulin, mg/dL	13.0 (9.9, 17.2)	11.6 (8.8, 15.8)	0.004
Current cigarette smoker, %	8.1	5.1	0.181
Antihypertensive medications, %	25.9	21.4	0.251

^a^chi-square for categorical variables, *t*-test and Wilcoxon rank sum tests for continuous variables. Mean ± standard deviation, median (25th, 75th percentile) or %.

**Table 2:** Subclinical measures of cardiovascular disease by hormone therapy use.

	Former users (*n* = 197)	Current users (*n* = 294)	*P* value^a^
Inter-adventitial diameter, mm	6.94 ± 0.54	6.79 ± 0.52	0.001
Lumen diameter, mm	5.44 ± 0.48	5.31 ± 0.46	0.002
Intima media thickness, mm	0.70 (0.65, 0.77)	0.71 (0.65, 0.77)	0.900
Presence of plaque, %	30.5	29.6	0.838

^a^chi-square for categorical, *t*-test and Wilcoxon rank sum tests for continuous. Mean ± standard deviation, median (25th, 75th percentile) or %.

**Table 3:** Univariate linear regression showing the association of demographic and cardiovascular risk factors with common carotid artery diameter.

	Interadventitial diameter		Lumen diameter	
	*β* (Std error)	*P* value	*β* (Std error)	*P* value
Current HT use	−0.157 (0.048)	0.001	−0.132 (0.043)	0.002
Age, years	0.017 (0.008)	0.037	0.005 (0.007)	0.460
African American race	−0.187 (0.076)	0.014	−0.067 (0.068)	0.326
Systolic blood pressure, mmHg	0.007 (0.002)	<0.0001	0.003 (0.002)	0.049
Diastolic blood pressure, mmHg	0.003 (0.003)	0.410	0.001 (0.003)	0.796
Pulse pressure, mmHg	0.010 (0.002)	<0.0001	0.004 (0.002)	0.025
Body mass index, kg/m^2^	0.031 (0.006)	<0.0001	0.021 (0.006)	0.0001
Weight, kg	0.013 (0.002)	<0.0001	0.009 (0.002)	<0.0001
Height, cm	0.016 (0.004)	<0.0001	0.012 (0.004)	0.001
Waist circumference, cm	0.009 (0.002)	<0.0001	0.007 (0.002)	0.001
Cholesterol, mg/dL	0.001 (0.001)	0.435	0.000 (0.001)	0.532
HDL cholesterol, mg/dL	−0.000 (0.002)	0.991	0.000 (0.002)	0.830
LDL cholesterol, mg/dL	0.001 (0.001)	0.338	0.001 (0.001)	0.396
Triglycerides, mg/dL	−0.000 (0.000)	0.631	−0.000 (0.000)	0.406
Glucose, mg/dL	0.009 (0.003)	0.001	0.006 (0.002)	0.013
Insulin, mg/dL	0.012 (0.004)	0.001	0.009 (0.003)	0.006
Current cigarette smoker	−0.205 (0.098)	0.037	−0.116 (0.088)	0.184
Antihypertensive medication use	0.132 (0.057)	0.020	0.088 (0.050)	0.083

Std: standard. HDL: high density lipoprotein. LDL: low density lipoprotein.

**Table 4:** Multivariable linear regression of factors associated with common carotid artery diameter.

	Interadventitial diameter		Lumen diameter	
	*β* (Std error)	*P* value	*β* (Std error)	*P* value
Current HT use	−0.137 (0.047)	0.004	−0.125 (0.043)	0.004
Age, years	0.013 (0.008)	0.106	0.003 (0.007)	0.630
African American race	−0.049 (0.074)	0.503	0.033 (0.068)	0.623
Pulse pressure, mmHg	0.008 (0.002)	0.0002	0.003 (0.002)	0.081
Weight, kg	0.012 (0.002)	<0.0001	0.009 (0.002)	<0.0001
Current cigarette smoker	−0.180 (0.093)	0.052	−0.106 (0.085)	0.214

Std: standard.
